# Benchmarking the HLA typing performance of Polysolver and Optitype in 50 Danish parental trios

**DOI:** 10.1186/s12859-018-2239-6

**Published:** 2018-06-25

**Authors:** Maria Luisa Matey-Hernandez, Danish Pan Genome Consortium, Lasse Maretty, Jacob Malte Jensen, Bent Petersen, Jonas Andreas Sibbesen, Siyang Liu, Palle Villesen, Laurits Skov, Kirstine Belling, Christian Theil Have, Jose M. G. Izarzugaza, Marie Grosjean, Jette Bork-Jensen, Jakob Grove, Thomas D. Als, Shujia Huang, Yuqi Chang, Ruiqi Xu, Weijian Ye, Junhua Rao, Xiaosen Guo, Jihua Sun, Hongzhi Cao, Chen Ye, Johan v Beusekom, Thomas Espeseth, Esben Flindt, Rune M. Friborg, Anders E. Halager, Stephanie Le Hellard, Christina M. Hultman, Francesco Lescai, Shengting Li, Ole Lund, Peter Løngren, Thomas Mailund, Maria Luisa Matey-Hernandez, Ole Mors, Christian N. S. Pedersen, Thomas Sicheritz-Pontén, Patrick Sullivan, Ali Syed, David Westergaard, Rachita Yadav, Ning Li, Xun Xu, Torben Hansen, Anders Krogh, Lars Bolund, Thorkild I. A. Sørensen, Oluf Pedersen, Ramneek Gupta, Simon Rasmussen, Søren Besenbacher, Anders D. Børglum, Jun Wang, Hans Eiberg, Karsten Kristiansen, Søren Brunak, Mikkel Heide Schierup, Søren Brunak, Jose M. G. Izarzugaza

**Affiliations:** 1 Center for Biological Sequence Analysis, Department of Bio and Health Informatics, Technical University of Denmark, DK-2800 Lyngby, Denmark; 2 Department of Twin Research and Genetic Epidemiology, Kings College London, London, UK; 3 Novo Nordisk Foundation Center for Protein Research, Faculty of Health and Medical Sciences, University of Copenhagen, DK-2200 Copenhagen, Denmark

**Keywords:** HLA genotyping, NGS, Clinical genomics, Population genetics, Prediction

## Abstract

**Background:**

The adaptive immune response intrinsically depends on hypervariable human leukocyte antigen (HLA) genes. Concomitantly, correct HLA phenotyping is crucial for successful donor-patient matching in organ transplantation. The cost and technical limitations of current laboratory techniques, together with advances in next-generation sequencing (NGS) methodologies, have increased the need for precise computational typing methods.

**Results:**

We tested two widespread HLA typing methods using high quality full genome sequencing data from 150 individuals in 50 family trios from the Genome Denmark project. First, we computed descendant accuracies assessing the agreement in the inheritance of alleles from parents to offspring. Second, we compared the locus-specific homozygosity rates as well as the allele frequencies; and we compared those to the observed values in related populations. We provide guidelines for testing the accuracy of HLA typing methods by comparing family information, which is independent of the availability of curated alleles.

**Conclusions:**

Although current computational methods for HLA typing generally provide satisfactory results, our benchmark – using data with ultra-high sequencing depth – demonstrates the incompleteness of current reference databases, and highlights the importance of providing genomic databases addressing current sequencing standards, a problem yet to be resolved before benefiting fully from personalised medicine approaches HLA phenotyping is essential.

## Background

The immune system is the forefront defence of higher organisms against disease. To perform its function, the immune system maintains a complex equilibrium between identifying a variety of external pathogens and recognising the organism’s own tissue. This process is carried out by the adaptive immune system [1–3]. The hallmark of the immune responses is the recognition of the offending antigen by the host cells through the major histocompatibility complex (MHC). In humans, it is known as the human leukocyte antigen (HLA) system and is located within a 3.6 Mb region on chromosome 6 (6p21.3) [3–5]. This region contains roughly 220 genes, which can be divided in HLA-like coding genes and non-HLA coding genes depending on their function and structure [6]. The accurate classification of the specificities of the HLA molecules based on their structural properties is still a matter of debate [7–9].

Traditionally, the HLA super-locus has been divided in five genomic sub-regions [10]. Within the encoded genes, further distinction is made between the so-called classical MHC genes, which encode the functional, epitope presenting molecules; and much less polymorphic, accessory non-classical genes [11, 12]*.* The class I classical MHC genes HLA-A, HLA-B and HLA-C, are expressed in all nucleated cells and are known to bind proteins from intracellular invading pathogens [13, 14]. The class II region, meanwhile, encodes α and β chain genes of the HLA type II dimers. These are primarily expressed in the so-called professional antigen-presenting cells (dendritic cells, macrophages and B cells) and have evolved to recognize exogenous proteins. The dimeric nature of the functional MHC-II complex and the copy-number variation of one of the loci (HLA-DRB) make this region particularly complicated [15]. The last gene-rich region, the class III region, encodes other conserved non-HLA genes with immune related functions. Cytokines represent a characteristic example of this category [6]. There are two additional regions, extended class I and extended class II, whose contributions to the gene count are minimal and are often disregarded [10]. Typically, for transplantation purposes only the classical genes are tested, there is an ongoing discussion on the role of non-classical genes in transplantation failure [11, 12]*.*

The hypervariability of the HLA region is key to the detection of a wide variety of pathogens and the activation of a cascade of defence mechanisms [10]. Owing to the selective pressure associated with immune functions, linkage disequilibrium patterns and allele frequencies are highly differentiated across populations [16]. These genes are segregated as a haplotype in a Mendelian fashion, making them suitable for population studies, as specific gene patterns and haplotypes are characteristic of geographic regions [17].

Several studies have related the variation of the HLA region to different diseases, including cancer [18] and type I diabetes [19]. As already mentioned, the region is a key determinant in the success of transplantation; allotransplants depend on equivalent HLA serotypes between individuals when no syngenic organ is available to avoid immune rejection of the organ [20]. All these scenarios need an accurate characterization of HLA genes. HLA typing, the process of addressing the HLA of an individual, is hindered by the complexity and hypervariability of the HLA region as discussed above [21]. Previous efforts [22–25] have tried to overcome the problems by matching with high accuracy those domains that directly interact with the antigen (exons 2 and 3 for HLA class I, and exon 2 for HLA class II), but this approach has proven to be insufficient alone [26]. Furthermore, while HLA typing and HLA gene validation are done routinely through molecular genotyping methods [27–29], the large and rapidly growing number of described HLA alleles are rendering them obsolete and unable to meet current clinical and research throughput demands [14, 30].

Automatic typing using computational methods has arisen as a possible solution to the expensive and time-consuming genotyping methods. Bioinformatic methods are more affordable than their experimental counterparts and benefit greatly from the constant development of algorithms in the field of computational genomics.

Methods for automatic typing of HLA regions are normally divided into assembly or alignment-based methods, according to whether the sequencing reads are either aligned to a reference, or the true alleles are predicted through probabilistic models [31]. Examples from both categories have been extensively benchmarked using curated data [31–33]. These data sets, which are considered gold standards, are obtained by PCR amplification using either already known alleles as primers or through Sanger sequencing, and then compared against an HLA database for designation. However, the relation between the gold standard and the database content presents problems. First, there is a large overlap between benchmarking cohorts [31]. Second, although they are useful for comparison of methods, the results obtained do not reflect the potential behaviour of the methods with different samples, especially if the new cohorts present different genetic backgrounds.

Two methods consistently produce the most accurate predictions of HLA typing: Optitype [25] and Polysolver [33]. Optitype identifies reads that map to exons 2 and 3 of the HLA class I alleles to select the most likely HLA class I allele from a custom database. Similarly, Polysolver [33] relies on a Bayesian probabilistic model to reassign reads that failed to map to the consensus reference genome. Here we present an analysis of the performance of two different alignment-based methodologies to characterize HLA type I alleles. We predict HLA alleles from high-depth, high-coverage sequencing data from a cohort of 50 Danish trios (father, mother, and child) in the context of the Genome Denmark project [34, 35]. The Genome Denmark project on these data has included, among other analysis, novel variation discovery [36] and therefore will not be covered in this publication. The Danish population is quite homogeneous and shows overall genomic resemblance to neighbouring countries [35]. This admixture is coherent with the history of the country [37]. Due to the quality of the assembly, this cohort constitutes a relevant resource for testing the robustness of the methods and for evaluating the effective coverage of the reference databases. Two different metrics evaluate different aspects of the accuracy of allele imputation. Our results validate the performance of the typers with a genetically different cohort, and reflect the importance of extending the current databases to achieve a better accuracy, with the prospect of using these methods in current medical practice. Finally, we compare the Danish population to other neighbouring countries by calculating the homozygosity index and the allele frequency suggesting a more precise estimate of the overrepresentation of certain HLA profiles within the Danish population.

## Methods

### HLA nomenclature and typing format

The WHO Nomenclature Committee for Factors of the HLA System have published 19 major reports to date, documenting HLA antigens, genes and alleles in response to the necessity for a systematic nomenclature for the polymorphic genes encoded in the HLA region. New alleles receive a unique identifier in the IPD-IMGT/HLA database (see below) after careful curation and analysis. These identifiers are composed of up to four sets of two digits separated by colons. The number of sets provided is often referred to as resolution. At the lowest resolution, only the first set of digits is provided (2-digit resolution), whereas a refined characterization of an allele would contain four sets (8-digit resolution), say HLA-A*02:01:01:02 L, that is an example of a full resolution allele. The first set of digits (HLA-A*02) defines the allele group as defined by a serological study of the antigen carried by the allele. The second set of digits (HLA-A*02:01, 4-digit resolution) defines an ordinal indicating the sequential order in which different subtypes were discovered. The third set of digits defines synonymous exonic variants (6-digits, HLA-A*02:01:01). Finally, the highest resolution level corresponds to alleles harbouring variants in untranslated regions such as those in introns, or in the 5′ or 3′ UTRs (8-digits, HLA-A*02:01:01:02). There are additional optional suffixes to an allele to indicate its expression status, such as low (L) expression or null (N) expression (not considered in the analyses presented here).

### Reference databases

#### IPD-IMGT/HLA

The IPD-IMGT/HLA database is part of the International ImMunoGeneTics (IMGT) databases. It contains sequences of the human major histocompatibility complex (MHC) and includes the official sequences named by the WHO Nomenclature Committee for Factors of the HLA System. This database contains 16,933 sequences and annotation information according to its latest version report (3.28.0 of 2017-04), In addition to the version report, monthly HLA Nomenclature updates are released, both in journals and online [38].

#### Allele Frequency Net

The Allele Frequency Net database is currently maintained by the consortium of the NHS Trust and the University of Liverpool. It contains frequency information of several immune genes such as Human Leukocyte Antigens, Killer-cell Immunoglobulin -like Receptors, and cytokines. Depending on the polymorphism, it contains population frequencies at the allele, haplotype or genotype levels [39].

#### Common and Well-Documented Alleles

The Common and Well-Documented (CWD) alleles’ catalogue is supported by the National Marrow Donor Program (US) and by Anthony Nolan (UK). The aim is to identify subsets of HLA alleles for which the frequencies are well known or have been validated multiple times through sequencing-based typing methods. Alleles are considered common when the frequency is observed to be greater than 0.001 in reference populations of at least 1500 individuals and reported more than three times in unrelated individuals, respectively. Currently, this catalogue is used in the National Marrow Donor program as reference for rare alleles [40].

### HLA typing methods

#### Optitype

Optitype works under the premise that the correct genotype is the one that explains the source of more reads than the rest of the genotypes. Hence, it finds the allele combination that maximizes the number of explained reads. Optitype overcomes the limitations of previous typers concerning ambiguous read alignment and suboptimal performance due to the exclusion of intronic information. For this, the custom database against which the reads are mapped contains genomic information that is limited to exons 2 and 3 together with small flanking intronic regions reconstructed from partially sequenced alleles with small phylogenetic distances. Although this database aims at improving typing, the resolution is limited by design to 4-digit resolution by the lack of extended genomic information. Also, the method currently has only HLA class I reference information readily available.

#### Polysolver

Polysolver is based on the reasoning that the coverage at HLA regions can be improved by identifying reads that failed to align to the canonical reference due to the accumulation of variants in these very hypermutable regions, and performing a realignment of such reads against a library of all known HLA alleles in the IPD-IMGT/HLA database. Thus, Polysolver enables high-precision HLA typing and mutation detection using the inferred alleles as a basis for said mutation. The method adopts a Bayesian classification approach where the allele with the highest probability is stored as the first correct allele and in a later iteration, the probabilities are recalculated taking into account the results from the previous search and the fact that the individual can be either heterozygous or homozygous [33]. Polysolver provides full resolution of up to 8-digits.

#### Allele reduction

The two aforementioned methods provide different resolution levels. For comparison purposes, alleles must present an identical resolution level. We converted identifiers of higher resolution than 4-digits using an allele reduction step. This process eliminates the excessive left-most pairs of numbers of the identifier, under the assumption that 6-digit and 8-digit resolutions describe variation of the same protein allele described at the level of 4-digits. For example, an allele A*02:01:01:02 would be converted to A*02:01 after allele reduction [41].

### The Genome Denmark cohort

The Genome Denmark cohort consists of 150 Danish individuals arranged in 50 trios (father, mother and child) [35]. Whole genome sequencing of these individuals was performed using Illumina technology at BGI Europe in Copenhagen, with an average depth of 80X and read length of 100 bp. Importantly, for each sample paired-end/mate-pair libraries were generated at different insert sizes of 180 bp, 500 bp, 800 bp, 2000 bp, 5000 bp, 10,000 bp, and 20,000 bp allowing for high quality assemblies, also of the highly polymorphic HLA region.

Trios in the Genome Denmark cohort were examined for their familial relationships. The HumanCoreExome BeadChip v.1.0 was used to genotype the trios using the HiScan system (Illumina, San Diego, California). Genotypes were called using GenomeStudio software (version 2011.1; Illumina). All subjects presented a high call rate above 98% and all familial relationships were confirmed.

Members of two families failed to map to the database used by Optitype and were therefore removed from the initial analysis. Thus, our analysed cohort consists of 48 out of the initial 50 family trios.

### HLA typing accuracy

To assess the confidence of the previously described methods for the Genome Denmark cohort, two different measures were defined for comparison. The Descent Accuracy (DA) is defined as the number of alleles of the progeny that can be explained by the typing of the parent’s alleles. DA is defined as follows:

3.1 $$ DA=\frac{N_{eq}}{N_{Alleles}}\kern1.5em $$

where *N*_*eq*_ is the number of alleles from the offspring that are coherently explained by the inheritance from the parents, and *N*_*alleles*_ the number of total alleles as described in (Eq. 3.2):

3.2 $$ {N}_{Alleles}={N}_{children}\ast 2\ast Locus $$

where *N*_*children*_ is the number of children in the population, and *Locus* is the number of loci to test. Each child carries two alleles per locus, one from each progenitor. For the complete HLA class I region, comprising three loci (HLA-A, -B and -C), every individual from the offspring would carry three times two alleles.

The Method Agreement (MA) measures the agreements between the two prediction methods. MA is defined as the number of identical alleles typed by the different methods:

3.3 $$ MA=\frac{N_{Optitype}={N}_{Polyso\mathrm{l} ver}}{N_{Population}\ast 2\ast Locus} $$

Extremely low MA values would indicate that the alleles tested differ enough from those of the database as for the typers to not agree in the imputed allele. On the other hand, high MA values as a measurement of identically typed alleles would mean an accurate representation of the alleles in the database used.

### Population analysis

We initially produced an overview of the population where firstly, the homozygosity ratio was compared with the homozygosity rates in the general population. This measurement is important because it is directly related to the runs of homozygosity (ROH) [42], which are regions of the genome that are identical despite having been inherited from both mother and father. The existence of these ROH can be explained by intermarriage, isolation and bottleneck situations, because the outcome of them is usually consanguinity. A high homozygosity rate can have medical consequences [43].

The homozygosity rate (HR) for alleles is described as follows:

3.4 $$ HR=\frac{N_{Hl}}{N_{Population}} $$

where *N*_*Hl*_ is the number of homozygous individuals in locus *L*. In this case, as the homozygosity implies a certain composition of the population, HR was tested for parents. The genetic background of the parents is undefined and therefore, their alleles and their frequency, are representative of the population. This is not the case for the children, if their possible alleles are a small subset defined by the alleles of the parents.

Then the allele frequency for each allele was calculated using the direct counting method [44]. For measuring the similarity with similar populations in terms of size and geographical proximity, the computed frequencies were compared to the information gathered in the two databases “Allele Frequency Net” and “Common and Well Documented Alleles” [39, 40].

## Results

### HLA typing

Here we used Optitype and Polysolver to type the individuals in the 50 family trios in the Genome Denmark cohort (150 individuals). HLA haplotypes are inherited in a Mendelian manner where the presence of each of the two alleles observed in the children must be explained by the presence of the same allele in either the mother or the father. The disposition in trios facilitates the traceability of the inheritance from parents to children. Due to typing problems, two families were discarded for the following analysis. Table 1 compares the descendent accuracy (DA) achieved by Optitype and Polysolver at different resolutions. DA indicates the fraction of alleles explained by direct inheritance from the parents.

**Table 1:** Descent Accuracy (DA) for the two typers considered

Typer	Overall	HLA-A	HLA-B	HLA-C
Polysolver (4-digit)	0.95	0.95	0.95	0.96
Optitype (4-digit)	0.88	0.95	0.82	0.87
Polysolver (8-digit)	0.64	0.47	0.68	0.77

Optitype at 4-digit resolution performed better than Polysolver having 8-digit resolution. However, when allele reduction is applied Polysolver surpasses the results provided by Optitype on the Genome Denmark cohort

When predictions across all alleles (HLA-A, HLA-B and HLA-C) are considered, Optitype produces coherent results between parents and children (0.88) across the 144 individuals in the Genome Denmark cohort. This is especially clear from the almost perfect (0.95) transmission of the predicted HLA-A alleles. The other two loci, HLA-B and HLA-C, follow closely with a DA of 0.82 and 0.87, respectively.

Contrarily, Polysolver produces predictions at its default 8-digit resolution that do not always transmit coherently from parents to children. DA ranges from 0.47 to 0.77, with an overall coherence of 0.64. These results might in part be explained by the increased number of possible alternatives due to the higher resolution and secondly to differences in complete, well assembled genomic regions rather than exons as is the case for Optitype.

To correct for the differences in resolution between the two methodologies, 8-digit typing results were collapsed into their 4-digit counterparts using the allele reduction protocol described in Methods. After allele reduction, Polysolver (4-digits) produced DA results that outperform those produce by Optitype. Overall, DA rises to 0.953. The largest improvement was observed for the HLA-A locus, where DA reached 0.95, which constitutes a 2-fold improvement. HLA-B and HLA-C followed with a final DA of 0.95 and 0.96, respectively. Both Polysolver and Optitype achieved similar HLA-A DA. Mismatched alleles often belong to the same serological groups (2-digits) than the correct types, in concordance with observations by existing benchmarks in spite of the different evaluation approaches implemented [32]. In our case, we evaluate the successful transfer of the serological group from parents to offspring while Kiyotani et al. compare against experimentally determined HLA alleles. Examples of incorrectly predicted alleles that still lay within the same serological group can be found in families 918 and 651 for Optitype, and families 1009 and 1030 for Polysolver, 8-digits. Interestingly, we find that HLA-B alleles still represent a challenge. This is also in agreement with previous observations [32]. In contrast to existing analyses [31, 32], our results suggest that Polysolver outperforms Optitype not only in the HLA-B region, which is the most polymorphic and a priori the most difficult to type, but also in its HLA-C counterpart. This improvement may stem from the different databases implemented by the methods; as the correct allele would likely only be present in the most complete database (Polysolver). Any small differences in the alignment against a restricted database such as the one implemented in Optitype would lead to incorrect typings.

As method-biases would affect all members of the family in the same manner, high DA is not necessarily equivalent to consistent predictions across two or more HLA typing methods. This effect is aggravated by the fact that the non-inherited alleles are not evaluated. To bridge this gap, we calculated an alternative statistic, which we refer to as method agreement (MA), to compare the complete set of predicted alleles between the two methods. MA provides good grounds to evaluate consistency in the predictions involving related individuals.

**Fig. 1:**
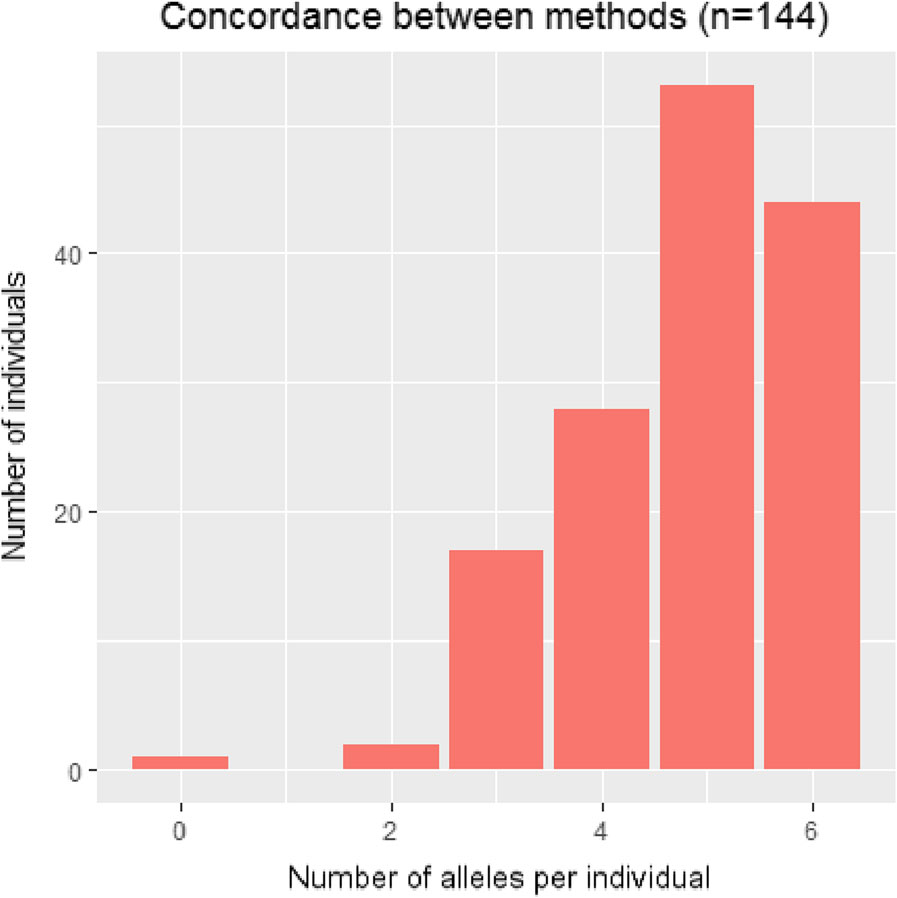
Concordance between the two methods at the level of individuals. The y-axis indicates the number of individuals, while the x-axis shows the number of alleles per individual identically typed for Optitype and Polysolver 4-D)

Typing accuracy across the methods (Polysolver and Optitype, both at 4-digit resolution) was evaluated in terms of MA. Overall, 63% of the alleles were congruently typed by both methods (Table 2). Interestingly, there were differences between the loci; HLA-A alleles were the most correctly predicted alleles, followed by HLA-B and HLA-C alleles. Furthermore, the majority of the individuals were typed either with complete concordance (6 identical alleles, 2 for each of the HLA-A, HLA-B and HLA-C loci) or with one discordant allele (Fig. 1). It is important to note that overall MA is mostly affected by several families rather than individuals (Fig. 1). Interestingly, we noticed that Polysolver had incorporated homozygous loci in almost all the wrongly typed cases. One particularly odd family was not only inconsistent between parents and offspring, but also inconsistent between the methods. In this case, Polysolver added many more homozygous sites than in other correctly typed loci. It is also worth noting how the discrepancies in the DA differ between the methods. In Optitype, the alleles wrongly typed according to the DA method are, in all the cases, wrongly typed in their entirety: not only do they not match at the allele level, but they also have the wrong serotype (2-digits level). This is also the case with Polysolver with full resolution and after allele reduction.

**Table 2:** Method agreement (MA) across the different loci and overall

	Overall	HLA-A	HLA-B	HLA-C
MA_TOTAL_	0.63	0.65	0.60	0.62
MA_T_	0.62	0.63	0.60	0.64
MA_NT_	0.63	0.67	0.60	0.60

MA represents the fraction of coherent alleles between Optitype and Polysolver at 4-digit resolution. MA_TOTAL_ refers to the complete set of alleles. MA_T_ refers to the portion of alleles that are inherited from parent to child, and MA_NT_ to those those that are not inherited and therefore, not part of the DA calculation

In family 1113, the alleles from HLA-A from the children can be explained by the parents, but the alleles inferred do not contribute to the MA as long as the methods imputed different alleles, and these alleles share neither 4- or 2-digit resolution. This is the case in several other families (1426 and 714). For this, although the typed alleles might be correct according to the DA, they should not by definition be seen as contributing to MA.

**Fig. 2:**
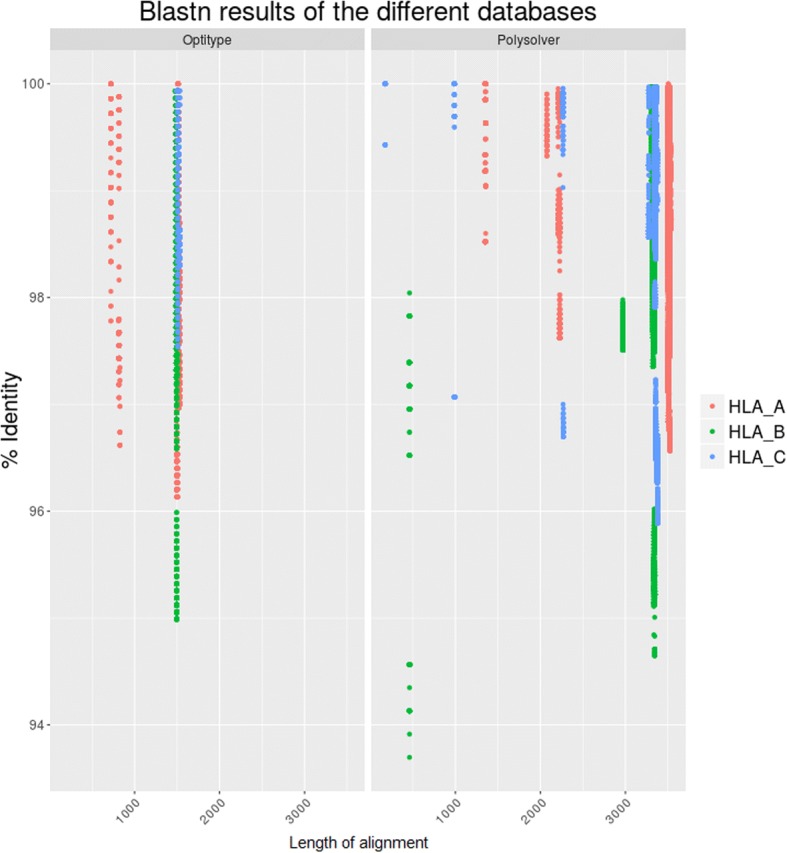
Blastn results of Optitype sequences against Optitype database (l) and Polysolver sequences against Polysolver database (r). In the plots we can observe that the identity within Optitype is higher than within Polysolver. This stems from the nature of the database. Optitype relies on a database with exons 2 and 3 and reconstructed introns, which produces sequences with scarce variation. As expected, Polysolver, due to including genomic sequences, has more variance in the identity within sequences. The self-blasted results (i.e. Sequence A against itself) were removed from the analysis

One could argue that a source of error is the presence of highly similar sequences in the database that would represent a challenge for the methods to discern. In the extreme case where two sequences are almost identical to the genomic allele of interest, the choice would be completely spurious and lack any biological information. Such cases should count as reduced error. We assessed the similarities between the sequences represented in each reference database with BLAST. For each allele considered by Optitype and Polysolver, we annotated the identity to any other sequence in their respective databases as provided by the method providers in the method installation packages (Fig. 2). Optitype presents a larger amount of sequences that align with more than 95% identity to other sequences of comparable length in its own database than Polysolver. These results fit within the description of the databases. Optitype sequences span exons 2 and 3, and reconstructed intronic regions, that are susceptible to be more similar than the whole genomic region. This highlights the importance of genomic databases instead of exonic, as the probability of incorrectly imputed allele is higher in the latter, simply for similarity reasons.

### Population analysis

The homozygosity rate accounts for the number of identical alleles in the same locus. This ratio is usually high in small, isolated populations due to poor genetic admixture; some specific allelic variants have evolved separately in ancestral genomes and display nowadays a characteristic geographical profile.

We calculated homozygosity rates for the HLA alleles identified in the parents of the Genome Denmark cohort (Table 3). Results are highly coherent between methods and although several individuals might present differential individual typing, the population maintains its structure. Overall, homozygosity rates are comparable between the methods, albeit slightly higher after allele reduction for Polysolver results. However, there are significant differences between the different individual loci. For HLA-A loci, Polysolver with 8-digit resolution achieved the same homozygosity rate as Optitype, while it was increased for the Polysolver (4-digits). HLA-B presents the smallest variance among the methods after performing allele reduction. In spite of HLA-B being the most polymorphic, both typers reached similar homozygosity rates. Both methods reached the highest level of homozygosity in HLA-C loci, the least polymorphic according to published results to date.

**Table 3:** Homozygosity Rates between methods

Typer	Overall	HLA-A	HLA-B	HLA-C
Optitype (4-digit)	0.09	0.08	0.08	0.11
Polysolver (8-digit)	0.08	0.08	0.02	0.14
Polysolver (4-digit)	0.12	0.12	0.09	0.15

Homozygosity Rates between methods, based on the number of identical alleles in each locus, either HLA-A, HLA-B or HLA-C; or across all three (Overall)

In addition to homozygosity rates, we computed the frequency of the individual alleles. The frequency of HLA alleles, inherited from parents to offspring, is a powerful tool in population genetics due to the population-wise variation they display [45]. As can be seen from Table 4 in the columns corresponding to the Genome Denmark cohort, although the frequencies are slightly different, the proportions are quite stable between methods.

**Table 4:** Comparison of allele frequencies between different populations

								Genome Denmark	
Allele	Northern Ireland	Sweden (South)	Sweden (North)	Germany	England (North)	Basque Country	Scotland Orkney	Polysolver	Optitype
A*02:01	0.27	0.26	0.24	0.28	0.29	0.27	NA	[#1] 0.26	[#1] 0.24
A*01:01	0.20	0.09	0.08	0.15	0.21	NA	NA	[#2] 0.21	[#2] 0.22
A*03:01	0.14	0.25	0.31	0.15	0.14	NA	NA	[#3] 0.17	[#3] 0.17
A*24:02	NA	0.13	0.21	0.09	0.07	NA	NA	[#4] 0.09	[#5] 0.04
A*11:01	0.08	0.06	0.01	0.06	0.07	NA	NA	[#5] 0.04	< 0.04
A*23:01	0.01	NA	NA	0.023	0.02	0.02	NA	< 0.045	[#4] 0.05
B*07:02	0.17	0.19	0.19	0.12	0.15	NA	NA	[#1] 0.18	[#1] 0.18
B*07:05	10^−3^	NA	NA	4 × 10^−3^	3 × 10^−3^	NA	NA	[#2] 0.15	< 0.06
B*15:01	0.04	0.14	0.15	0.06	0.06	NA	NA	[#3] 0.07	[#4] 0.09
B*44:02	0.13	0.10	0.03	0.07	0.10	NA	0.26	[#4] 0.07	[#3] 0.09
B*40:01	0.05	0.10	0.14	0.05	0.06	NA	0.06	[#5] 0.06	[#5] 0.06
B*08:01	0.16	0.07	0.05	0.09	0.15	NA	0.17	< 0.06	[#2] 0.13
C*07:01	NA	NS	NS	0.15	0.19	NA	NA	[#1] 0.19	[#1]0.2
C*07:02	0.19	NS	NS	0.13	0.16	NA	NA	[#2] 0.18	[#2] 0.17
C*06:02	0.09	NS	NS	0.1	0.09	0.03	0.07	[#3] 0.11	[#4] 0.1
C*03:03	0.05	NS	NS	0.05	0.06	0.07	0.09	< 0.08	[#3] 0.11
C*03:04	0.06	NS	NS	0.07	0.08	0.05	0.05	[#4] 0.09	[#5] < 0.09
C*05:01	0.13	NS	NS	0.06	0.10	0.14	5 × 10^−3^	[#5] 0.08	0.09

Comparison of allele frequencies between different historically related populations through settlements (Northern Ireland, England, Scotland Orkney), geo-graphically nearness (Sweden, Germany) and not related (Basque Country). The “NA” value means that the particular allele is either not present in the population or not significant. “NS” means there is no data for this allele in the corresponding study. The top five most frequent alleles for each loci per method are included for the Genome Demark cohort. The alleles marked with “#” indicate the order of said allele in the ranking of the most common alleles

Compared to the expected allele frequency database values, there are some discrepancies in the proportions regarding the populations. While the most common allele for HLA-B is B*07:02 and indeed is the most common in the Caucasian population according to the database, the rest of the alleles typed by these methods are rarely seen. For example, the second most common allele in the Danish population according to Polysolver, B*07:05, is not even present in a relevant proportion in other related populations, where it is seldom observed (Table 4). B*07:05 is also inconsistent between the analysed methods, where Optitype seems to favour B*08:01 instead. Upon reviewing the database used, we observed that the B*08:01 allele is frequently represented, due to extensive intronic reconstruction, whereas B*07:05 only has six possible alleles. The mapping would naturally fail in examples where differences would lie within the other exonic/intronic regions or where the reconstructed regions were largely different to the real allele. In this case, if B*07:05 and B*08:01 are similar, the probability of Optitype wrongly aligning to either of them depends on the number of available alleles and the similarities among them.

We compared our results to those for known populations in the database of Common and Well-Documented alleles [40]. For all the loci, the most common alleles are those common to other historically connected populations such as North Ireland, Sweden and Norway. This comparison considers sequence similarity but also similar proportion in the population [46]. The rarer alleles, however, have larger than expected proportions in our cohort, suggesting that some of these alleles could indeed be population specific.

Locus HLA-A is the one with most similarities among closely related populations. The most common allele in the Danish population according to both typers (A*02:01) is also commonly found in populations within the geographical proximity to Denmark, including Sweden and Germany. This allele is also very prevalent in other more distant populations. Similar proportions for the least represented alleles are found across populations. Interestingly, the second most common allele (A*01:01) has a frequency closer to historically related settlements in Northern Ireland and England than to countries with shared borders and similar genetic background. The third most common allele (A*03:01) has a frequency dissimilar to any other and probably reflects the homogeneity of the Danish population. HLA-C alleles also suggest similarities to the frequencies found in Northern Ireland and England, which harboured known Viking settlements, rather than to the countries in the geographical vicinity.

Again, the most dissimilar locus is HLA-B, and the one where the biggest fluctuation of alleles is found. It can be seen in the proportion of the most common alleles: HLA-B has less homogeneity in which alleles are in the population, as if no allele HLA-B has been fixed. This can either mean that the typing is wrong, which could be the case regarding all previous results, or that the HLA-B has not been decisive for the population.

**Fig. 3:**
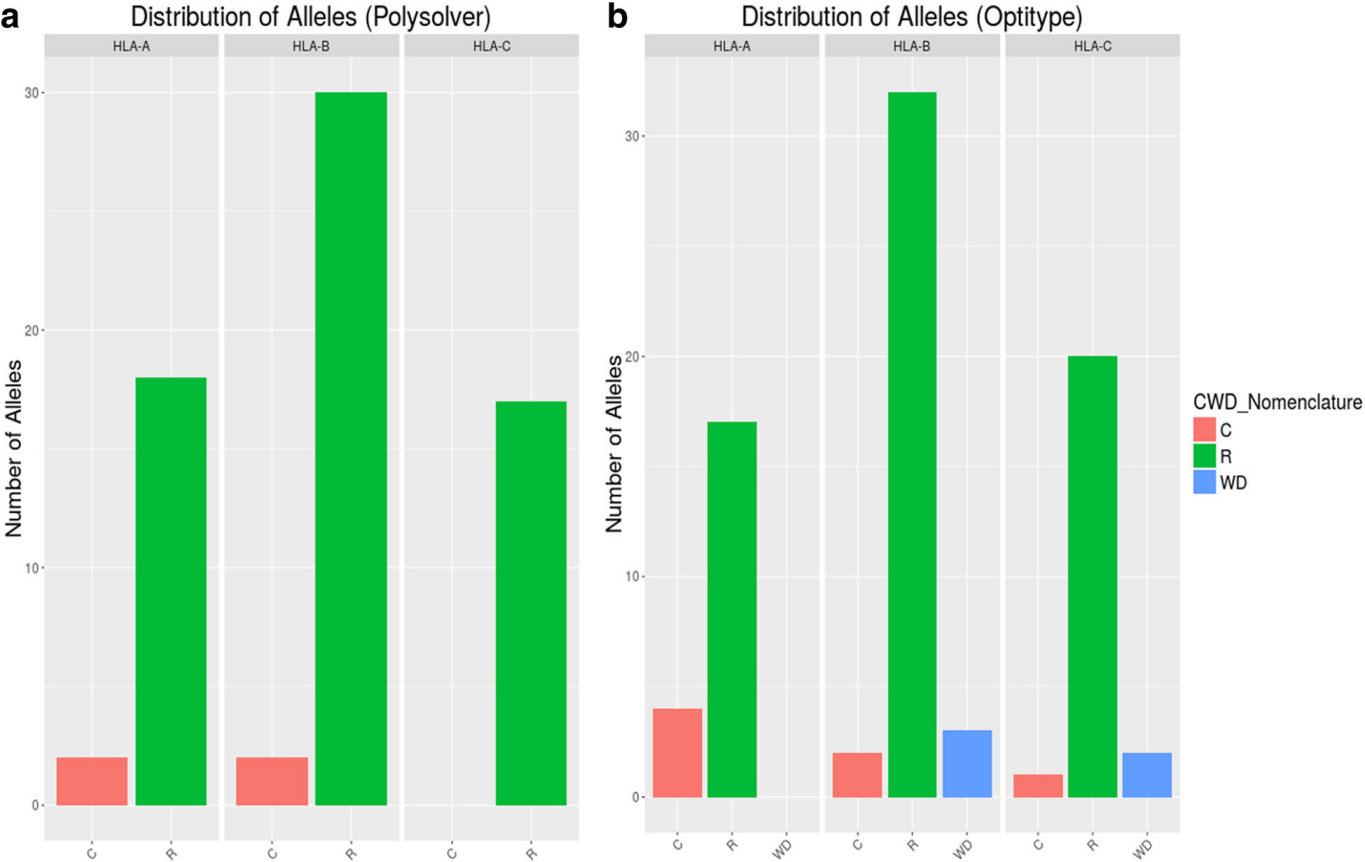
Distribution of alleles according to CWD for Polysolver (**a**) and Optitype (**b**). These results highlight that HLA- B harbours the rarest alleles

Following the results above, if the alleles were indeed rare, they should be indicated as such in the Common and Well-Documented alleles database. The variety of different alleles typed by Optitype is less significant than for Polysolver. Among the methods, HLA-A and HLA-C were most similar to other populations in terms of allele distribution. In Fig. 3, both loci have some alleles that are common (those that match other populations) but they also display several rare alleles, those less frequent. As expected, HLA-B has the highest rate of rare alleles, which confers value to the results found in the homozygosity rate analysis.

## Discussion

We have performed here a comprehensive analysis of the HLA region in the Genome Denmark cohort, using two of the available bioinformatics methods, Optitype and Polysolver. Our results show that in general, the two HLA-typers compared reasonably well. Both methods yielded an accuracy higher than 80% as observed in previous studies [22–24]. Our analysis yields results that differ from existing benchmarks by Kiyotani et al. and Bauer et al. We propose Polysolver as the most accurate typer for 4-digits resolution. Bauer et al. do not consider Polysolver in their analysis due to technical limitations. Kiyotani et al. perform an analysis on 12 clinical samples, whereas the analysis presented here relies on 50 Danish parental trios. The increased sample size may represent more accurately the diversity of existing alleles.

Moreover, the study presented here is, to our knowledge, the first to compare WGS data between these two typers. Both existing benchmarks evaluate comparisons stemming mainly from WES data with a theoretical 4-digit resolution upper limit. Bauer and collaborators expand further this limit to 6-digit and 8-digit resolution by including RNAseq and simulated data, respectively [31]. Nevertheless, using high-depth WGS data in combination with family information, provides in our opinion, two fundamental advantages: First, the agreement between parents and children offers accuracy estimations independent from the availability of curated gold standard data. Second, HLA sequences are considered in their whole extent, including intronic regions. These are typically disregarded by other methods with reduced accuracy in spite of their plausible functional relevance.

In terms of performance, the correct choice of a reference database remains as the main challenge. Probabilistically, larger databases are in disadvantage against limited databases in terms of assessing correctly among the alleles represented. Despite this consideration, Polysolver produces better results after reduction of the resolution to levels comparable to those produced by Optitype. Furthermore, it can be argued that the typing from Polysolver, as derived from a more complete database, is more reliable, as Optitype in its simplicity might not be typing the right allele.

The main drawback of Optitype is that it relies on a curated database that mainly contains exons and a limited number of flanking introns. A genomic region can be fairly similar to another one in the exonic part, especially if it belongs to the same serotype, but differ greatly in several other parts of the sequence. This would hinder the comparison between methods that provide different default resolutions: while Polysolver uses a statistical model, Optitype applies a simpler alignment-based method. Polysolver in its highest resolution also gives an insight into the importance of having the most complete database. In general, and considering that the benchmarked methods were the currently best ones, we can say that no available method is accurate for the highest level of resolution.

Homozygosity ratios are also useful for describing a population. In principle, the high homozygosity imputed to this cohort can be explained by the cohort itself, as the individuals have been chosen to be representatives of an ancestral Danish population. In our results, the homozygosity ratio provided by Polysolver is higher after allele reduction (4-digits) than when using 8-digits resolution. These results are as expected, especially if the differences between alleles are exclusively located in intronic regions. For Optitype, the results are very similar. This has biological relevance. The 4-digit resolution at the protein level, includes differences in protein-coding regions. In homogeneous populations, the advantageous alleles are fixed. In HLA, the advantage of an allele lies in the binding groove in exons 2 and 3, so similar homozygosity ratios from both typers are expected. Interestingly, the most homozygous locus, HLA-C, is also the locus with the lowest MA. These two discrepancies together might indicate that there is no correct allele in the database for the HLA-C alleles of the cohort, but very similar ones only.

With the population analysis, we addressed the genetic similarity between populations. In general, Danes resemble historically related population in the frequency of the most common alleles, but not to countries in the geographical vicinity. It is more noticeable for HLA-B, where the difference between the most common allele and the rest are larger than in any of the other loci, in addition to the differences between methods. These differences can be largely due to the representation of the alleles in the database. For Optitype, B*08:01 is overrepresented in comparison with B*07:05. As the Optitype method of imputation is based on the number of reads mapped to a specific allele, a higher number of alleles from the same 4-digits group increases the probability of overrepresentation.

## Conclusion

HLA genetics is as complex as it is useful. The HLA region is important not only for transplantation, but it has also been related to a myriad of autoimmune diseases and cancer and used in many other research fields such as population genetics. The usefulness of the genes in this part of the genome is directly related to our ability to identify correctly the alleles that each individual has. So far, the molecular genetics methods have been the gold standard, but the recent advances in sequencing and bioinformatics approaches can shift views towards what not also emerges in the personalized medicine field.

These approaches, though, are still in their infancy. While HLA class I alleles are less complex to type, they have been extensively used as proof of concept for different typing approaches as the ones compared here. In spite of that, only Polysolver has achieved an accuracy similar to those already reported in the previous benchmarks, indicating that there is still room for improvement in the field. Previous benchmarks highlighted the importance of larger, more diverse databases. Our results in a distinct homogeneous population are coherent also support that view. Also, if these methods aim for being used in clinic, future tools need to incorporate the HLA class II region, and probably, HLA class III. The current methods, however imperfect, are a step in the right direction. Although the accuracy is not as high as previous authors have claimed using with the gold standard, the studied typers are somewhat robust, as they have managed to type accurately a different cohort, which holds new variation within their genetic sequences [34].

Despite the robustness, the amount of new data and alleles being added every day to the database, new GWAS and novel studies about mismatch in donor organs are leaving the 4-digits typing obsolete. Current methods have problems of underperformance when dealing with the ever-expanding list of alleles, as we and other researcher have brought forward bioinformatics efforts and whole genome sequencing cohorts like Genome Denmark are an invaluable source of information for these databases. Similar efforts in isolated or geographically remote populations are an interesting field of research, and the information from them important if these techniques are to replace PCR-based methods.

In conclusion, assuming that the quality of reference databases increases steadily in the future, algorithmic changes are urgently needed. The rapid growth of the number of alleles, the new NGS methods and the new studies disregarding the acceptable mismatches for organ donation, typers should include the whole database of available HLA alleles [47], and better methods of imputation.

## Abbreviations

CWD: Common and Well-Documented
DA: Descendent accuracy
HLA: Human leukocyte antigen
HR: Homozygosity rate
IMGT: International ImMunoGeneTics
MA: Method Agreement
MHC: The major histocompatibility complex
NGS: Next-generation sequencing
ROH: Runs of homozygosity

## References

[1] Wilson J, Hunt T (2002). T cells and MHC proteins. Molecular biology of the cell.

[2] Iwasaki A, Medzhitov R (2015). Control of adaptive immunity by the innate immune system. Nat Immunol.

[3] Ayala Garcia MA, Gonzalez Yebra B, Lopez Flores AL, Guani Guerra E (2012). The major histocompatibility complex in transplantation. J Transp Secur.

[4] Fitch WM, Ayala FJ, National Academy of Sciences (U.S.) (1995). Molecular Genetics of Speculation and Human Origins. Tempo and mode in evolution: genetics and paleontology 50 years after Simpson.

[5] Trowsdale J, Knight JC (2013). Major histocompatibility complex genomics and human disease. Annu Rev Genomics Hum Genet.

[6] Horton R, Wilming L, Rand V, Lovering RC, Bruford EA, Khodiyar VK, Lush MJ, Povey S, Talbot CC, Wright MW (2004). Gene map of the extended human MHC. Nat Rev Genet.

[7] Doytchinova IA, Guan P, Flower DR (2004). Identifiying human MHC supertypes using bioinformatic methods. J Immunol.

[8] Lund O, Nielsen M, Kesmir C, Petersen AG, Lundegaard C, Worning P, Sylvester-Hvid C, Lamberth K, Roder G, Justesen S (2004). Definition of supertypes for HLA molecules using clustering of specificity matrices. Immunogenetics.

[9] Nielsen M, Lundegaard C, Worning P, Hvid CS, Lamberth K, Buus S, Brunak S, Lund O (2004). Improved prediction of MHC class I and class II epitopes using a novel Gibbs sampling approach. Bioinformatics.

[10] Shiina T, Hosomichi K, Inoko H, Kulski JK (2009). The HLA genomic loci map: expression, interaction, diversity and disease. J Hum Genet.

[11] Carlini F, Ferreira V, Buhler S, Tous A, Eliaou J-F, Rene C, Chiaroni J, Picard C, Di Cristofaro J (2016). Association of HLA-A and non-classical HLA class I alleles. PLoS One.

[12] Kochan G, Escors D, Breckpot K, Guerrero-Setas D (2013). Role of non-classical MHC class I molecules in cancer immunosuppression. Oncoimmunology.

[13] Garstka MA, Fish A, Celie PH, Joosten RP, Janssen GM, Berlin I, Hoppes R, Stadnik M, Janssen L, Ovaa H (2015). The first step of peptide selection in antigen presentation by MHC class I molecules. Proc Natl Acad Sci U S A.

[14] Lund O, Nascimento EJM, Maciel M, Nielsen M, Larsen MV, Lundegaard C, Harndahl M, Lamberth K, Buus S, Salmon J (2011). Human leukocyte antigen (HLA) class I restricted epitope discovery in yellow fewer and dengue viruses: importance of HLA binding strength. PLoS One.

[15] Neefjes J, Jongsma ML, Paul P, Bakke O (2011). Towards a systems understanding of MHC class I and MHC class II antigen presentation. Nat Rev Immunol.

[16] Evseeva I, Nicodemus KK, Bonilla C, Tonks S, Bodmer WF (2010). Linkage disequilibrium and age of HLA region SNPs in relation to classic HLA gene alleles within Europe. Eur J Hum Genet.

[17] Fernandez Vina MA, Hollenbach JA, Lyke KE, Sztein MB, Maiers M, Klitz W, Cano P, Mack S, Single R, Brautbar C (2012). Tracking human migrations by the analysis of the distribution of HLA alleles, lineages and haplotypes in closed and open populations. Philos Trans R Soc Lond Ser B Biol Sci.

[18] Stranzl T, Larsen MV, Lund O, Nielsen M, Brunak S (2012). The cancer exome generated by alternative mRNA splicing dilutes predicted HLA class I epitope density. PLoS One.

[19] Brorsson C, Tue Hansen N, Bergholdt R, Brunak S, Pociot F (2010). The type 1 diabetes - HLA susceptibility interactome--identification of HLA genotype-specific disease genes for type 1 diabetes. PLoS One.

[20] Sheldon S, Poulton K (2006). HLA typing and its influence on organ transplantation. Methods Mol Biol.

[21] Rees L, Kim JJ (2015). HLA sensitisation: can it be prevented?. Pediatr Nephrol.

[22] Boegel S, Lower M, Schafer M, Bukur T, de Graaf J, Boisguerin V, Tureci O, Diken M, Castle JC, Sahin U (2012). HLA typing from RNA-Seq sequence reads. Genome Med.

[23] Bai Y, Ni M, Cooper B, Wei Y, Fury W (2014). Inference of high resolution HLA types using genome-wide RNA or DNA sequencing reads. BMC Genomics.

[24] Kim HJ, Pourmand N (2013). HLA haplotyping from RNA-seq data using hierarchical read weighting. PLoS One.

[25] Szolek A, Schubert B, Mohr C, Sturm M, Feldhahn M, Kohlbacher O (2014). OptiType: precision HLA typing from next-generation sequencing data. Bioinformatics.

[26] Mahdi BM (2013). A glow of HLA typing in organ transplantation. Clin Transl Med.

[27] Tinckam KJ, Chandraker A, Sayegh MH, Singh AK (2012). Basic histocompatibility testing methods. Core concepts in renal transplantation.

[28] Dunckley H (2012). HLA typing by SSO and SSP methods. Methods Mol Biol.

[29] Schmitz JL, Coleman WB, Tsongalis GJ (2005). HLA typing using molecular methods. Molecular diagnostics: for the clinical laboratorian.

[30] La Manna G, Corsini S, Iannelli S, Cappuccilli ML, Comai G, Iorio M, Todeschini P, Carretta E, Scolari MP, Bontadini A (2013). Influence of the immunogenetic KIR and HLA systems on long-term renal transplant outcome. Ann Transplant.

[31] Bauer DC, Zadoorian A, Wilson LO, Melbourne Genomics Health A, Thorne NP. Evaluation of computational programs to predict HLA genotypes from genomic sequencing data. Brief Bioinform. 2016;19(2):179–87. 10.1093/bib/bbw097 PMC6019030 27802932

[32] Kiyotani K, Mai TH, Nakamura Y (2017). Comparison of exome-based HLA class I genotyping tools: identification of platform-specific genotyping errors. J Hum Genet.

[33] Shukla SA, Rooney MS, Rajasagi M, Tiao G, Dixon PM, Lawrence MS, Stevens J, Lane WJ, Dellagatta JL, Steelman S (2015). Comprehensive analysis of cancer-associated somatic mutations in class I HLA genes. Nat Biotechnol.

[34] Besenbacher S, Liu S, Izarzugaza JM, Grove J, Belling K, Bork-Jensen J, Huang S, Als TD, Li S, Yadav R (2015). Novel variation and de novo mutation rates in population-wide de novo assembled Danish trios. Nat Commun.

[35] Maretty L, Jensen JM, Petersen B, Sibbesen JA, Liu S, Villesen P, Skov L, Belling K, Theil Have C, Izarzugaza JMG (2017). Sequencing and de novo assembly of 150 genomes from Denmark as a population reference. Nature.

[36] Jensen JM, Villesen P, Friborg RM, Danish Pan-Genome C, Mailund T, Besenbacher S, Schierup MH, Maretty L, Jensen JM, Petersen B (2017). Assembly and analysis of 100 full MHC haplotypes from the Danish population. Genome Res.

[37] Athanasiadis G, Cheng JY, Vilhjalmsson BJ, Jorgensen FG, Als TD, Le Hellard S, Espeseth T, Sullivan PF, Hultman CM, Kjaergaard PC (2016). Nationwide genomic study in Denmark reveals remarkable population homogeneity. Genetics.

[38] Robinson J, Halliwell JA, Hayhurst JD, Flicek P, Parham P, Marsh SG (2015). The IPD and IMGT/HLA database: allele variant databases. Nucleic Acids Res.

[39] Gonzalez-Galarza FF, Takeshita LY, Santos EJ, Kempson F, Maia MH, da Silva AL, Teles e Silva AL, Ghattaoraya GS, Alfirevic A, Jones AR (2015). Allele frequency net 2015 update: new features for HLA epitopes, KIR and disease and HLA adverse drug reaction associations. Nucleic Acids Res.

[40] Mack SJ, Cano P, Hollenbach JA, He J, Hurley CK, Middleton D, Moraes ME, Pereira SE, Kempenich JH, Reed EF (2013). Common and well-documented HLA alleles: 2012 update to the CWD catalogue. Tissue Antigens.

[41] Marsh SGE (2016). Nomenclature for factors of the HLA system, update April 2016. Int J Immunogenet.

[42] Kirin M, McQuillan R, Franklin CS, Campbell H, McKeigue PM, Wilson JF (2010). Genomic runs of homozygosity record population history and consanguinity. PLoS One.

[43] Bittles AH (2001). Consanguinity and its relevance to clinical genetics. Clin Genet.

[44] Shen C, Zhu B, Liu M, Li S (2008). Genetic polymorphisms at HLA-A, -B, and -DRB1 loci in Han population of Xi’an city in China. Croat Med J.

[45] Sanchez-Mazas A, Meyer D (2014). The relevance of HLA sequencing in population genetics studies. J Immunol Res.

[46] McEvoy B, Brady C, Moore LT, Bradley DG (2006). The scale and nature of Viking settlement in Ireland from Y-chromosome admixture analysis. Eur J Hum Genet.

[47] Tinckam KJ, Rose C, Hariharan S, Gill J (2016). Re-examining risk of repeated HLA mismatch in kidney transplantation. J Am Soc Nephrol.

[48] Eiberg H, Nielsen LS, Klausen J, Dahlen M, Kristensen M, Bisgaard ML, Moller N, Mohr J (1989). Linkage between serum cholinesterase 2 (CHE2) and gamma-crystallin gene cluster (CRYG): assignment to chromosome 2. Clin Genet.

